# Workshop for Basic Gynaecological Examinations: Improving Medical Student Learning through Clinical Simulation

**DOI:** 10.3390/healthcare11162352

**Published:** 2023-08-21

**Authors:** Yolanda Cuñarro-López, Lucia Sánchez Llanos, Ignacio Cueto Hernández, Blanca González-Garzón De Zumárraga, María Del Pilar Pintado Recarte, Francisco Javier Ruiz Labarta, Óscar Cano-Valderrama, Olga Aedo Ocaña, Raquel Pérez Lucas, María Del Carmen Viñuela Benéitez, Zurine Raquel Reyes Angullo, María Fernández Muñoz, Juan Manuel Pina Moreno, Maria Mercedes Sanchez Rodriguez, Rocío Aracil Rodríguez, Laura Pérez Burrel, Ainoa Sáez Prat, Andrea Fraile López, Beatriz Gutiérrez Del Río, María de Guadalupe Quintana Coronado, Tamara Cisternas, Marta Feltrer Hidalgo, Pablo González Navarro, Miguel A. Ortega, Carlos López Ramón y Cajal, Juan Antonio De León-Luis

**Affiliations:** 1 Department of Public and Maternal and Child Health, School of Medicine, Complutense University of Madrid, 28040 Madrid, Spain; yolanda.cunarro.lopez@sergas.es (Y.C.-L.); lucsan07@ucm.es (L.S.L.); dr_icueto@yahoo.es (I.C.H.); bgonzalezg@salud.madrid.org (B.G.-G.D.Z.); ppintadorec@yahoo.es (M.D.P.P.R.); franciscojavier.ruiz@salud.madrid.org (F.J.R.L.); olgaedo@hotmail.com (O.A.O.); raquelplucas@hotmail.com (R.P.L.); mcvinuela73@hotmail.com (M.D.C.V.B.); zurineraquel.reyes@salud.madrid.org (Z.R.R.A.); maria_ferny2@hotmail.com (M.F.M.); juanmpinam@gmail.com (J.M.P.M.); mariamercedes.sanchez.rodriguez@salud.madrid.org (M.M.S.R.); rocioaracilrodriguez@gmail.com (R.A.R.); lauraperezburrel@gmail.com (L.P.B.); ainoa_saez@hotmail.com (A.S.P.); afrailelopez@gmail.com (A.F.L.); beatrizgutierrez95@hotmail.com (B.G.D.R.); guadalupe4qc@gmail.com (M.d.G.Q.C.); tamcisters@gmail.com (T.C.); marta.feltrer@gmail.com (M.F.H.); jaleon@ucm.es (J.A.D.L.-L.); 2 Department of Obstetrics and Gynecology, University Hospital Gregorio Marañón, 28009 Madrid, Spain; 3 Health Research Institute Gregorio Marañón, 28009 Madrid, Spain; 4 Department of Obstetrics and Gynecology, University Hospital Álvaro Cunqueiro, 36312 Vigo, Spain; carlos.lopez.ramon.y.cajal@sergas.es; 5 Department of Surgery, University Hospital Álvaro Cunqueiro, 36312 Vigo, Spain; oscar.cano.valderrama@sergas.es; 6 Methodology and Biostatistics Unit, Gregorio Marañón Health Research Institute (IiSGM), 28009 Madrid, Spain; pablo.gonzalez@iisgm.com; 7 Department of Medicine and Medical Specialities, Faculty of Medicine and Health Sciences, University of Alcalá, 28871 Alcalá de Henares, Spain; 8 Ramón y Cajal Institute of Sanitary Research (IRYCIS), 28034 Madrid, Spain

**Keywords:** gynaecological examinations, workshop, questionnaires, formation

## Abstract

Introduction: This study was designed to evaluate whether the Workshop on Basic Principles for Clinical Gynaecological Exploration, offered to medical students, improves theoretical–practical knowledge, safety, confidence, global satisfaction and the achievement of the proposed objectives in the area of gynaecological clinical examinations. Materials and Methods: This was a quasi-experimental pre–post-learning study carried out at the Gynaecology and Obstetrics department of Gregorio Marañón Hospital in Madrid (Spain). The volunteer participants were 4th-year students earning a degree in Medicine during the 2020–2021 and 2021–2022 academic years. The study period was divided into the following stages: pre-workshop, intra-workshop and 2 weeks post-workshop. In the pre-workshop stage, students completed a brief online course to prepare for the workshop. The effectiveness of the workshop was evaluated through multiple-choice tests and self-administered questionnaires to assess self-assurance, self-confidence, self-satisfaction and the achievement of the objectives. Results: Of the 277 students invited in both academic years, 256 attended the workshop (92.4%), with a total participation in the different stages of the study greater than 70%. A total of 82.5% of the students in the 2020–2021 academic year and 80.6% of students in the 2021–2022 academic year did not have any type of experience performing gynaecological clinical examinations. Between the pre-workshop and 2 weeks post-workshop stages, there was significant improvement in theoretical–practical knowledge (improvement mean = 1.38 and 1.21 in 2020–2021 and 2021–2022 academic years, respectively). The security and confidence of the students prior to the workshop were low (average scores less than 5 points) in both academic years. However, post-workshop scores for satisfaction and the achievement of objectives were high in the two academic years; all the values approached or exceeded 8 points. Conclusions: Our students, after outstanding participation, evaluated the BPCGE, and improved their theoretical and practical knowledge, as well as their skills in a gynaecological clinical examination. Moreover, in their view, after the workshop, they felt very satisfied, far outreaching the proposed aims. In addition, excellent results were maintained over time, year after year.

## 1. Introduction

The goal of undergraduate medical education is to provide students with a solid background in health sciences, in-depth knowledge of pathophysiology and pharmacology and the skills to perform medical diagnoses, carry out procedures and administer various treatments to patients [1]. Therefore, training is essential in medical education. Bridging the gap between theoretical knowledge and practical application, it gives students the abilities, exposure and values they need to succeed in the medical field. Students must actively participate in the acquisition of competencies, both at the clinical level and in the doctor-patient relationship. They can learn vital clinical abilities like how to conduct physical examinations, communicate with patients and perform medical procedures. Theoretical knowledge alone cannot sufficiently teach these skills. Students who study medicine are exposed to real-world situations that they will deal with in the workplace. Students obtain the chance to put their knowledge to use in real patient care settings through clinical rotations, internships and simulation exercises. They gain an understanding of the complexity of healthcare delivery thanks to this exposure, which also helps them become ready for the difficulties future healthcare workers will encounter. Moreover, job training will provide not only confidence and competence for the student but also interprofessional collaboration with various healthcare professionals with longer work experience.

Likewise, the information provided through a clinical examination especially in areas as sensitive as gynaecology and obstetrics is of vital importance, so ethical development is not less important to mention. Training emphasizes moral decision making, patient confidentiality, empathy and cultural awareness, resolves moral dilemmas, enforces professional boundaries and upholds the standards of patient care.

The modern era of clinical simulation in the teaching of medicine has been developing since the 1960s [2] and has already been implemented in the gynaecology and obstetrics environment [3,4,5,6]. While virtual clinical simulation cannot fully replace the hands-on experience gained through direct patient care, it can serve as a valuable complement to traditional learning methods in this medical specialty. It offers a safe, risk-free, interactive and immersive learning environment that allows for the acquisition and application of knowledge, skills (technical and nontechnical) and clinical decision-making attitudes by students in health sciences. Simulations recreate real situations in the workplace, promoting the systematization and repetition of processes in a safe environment [7]. In addition, they serve as training tools that reduce the probability of error, promote the ability to work under stress and strengthen teamwork and the skills acquired by health personnel, ultimately resulting in greater patient safety [3,8,9].

Currently, most universities and hospitals focus their interest on determining how their students, from undergraduate students to medical residents, nursing staff and medical specialists, learn in different training approaches. In addition, it is important to assess to what degree training has had an impact on the behaviour of different generations, both current and future, and how the results obtained have led to modifications [10]. High-quality images, 3D models and interactive elements are frequently provided with virtual simulations to aid in the visualization and comprehension of anatomical structures and operations. Students can handle objects, explore the virtual environment from various perspectives and learn more about the intricate details of gynaecological and obstetrical anatomy. Additionally, students can discover areas for growth and monitor their progress over time with the use of real-time feedback and evaluation elements in virtual simulations.

A controlled learning environment also supplies objective metrics to assess students’ progress. Completion time, procedure accuracy and adherence to best practices are a few examples of these metrics. Students are better able to identify their strengths and areas for development when their progress is objectively monitored and compared to benchmarks. This measurement-based feedback encourages self-evaluation and inspires pupils to work toward specific goals.

Since 2017, the Gynaecology and Obstetrics department of Hospital Universitario Gregorio Marañón (HUGM), Madrid, Spain has been holding a series of clinical simulation workshops for undergraduates of Medicine at the Complutense University of Madrid (CUM), aimed at improving their understanding of maternal–neonatal health [11]. Examples of the training workshops include the Outpatient Vaginal Delivery Workshop and the Blood Loss Quantification Workshop. In a study by Ruiz-Labarta et al., these workshops have been shown to improve, in an objective and verifiable way, the theoretical–practical knowledge, safety and confidence of assistants in various gynaecological–obstetric procedures [11]. 

Regarding simulations for different levels of gynaecology and obstetrics, we previously evaluated the degree of compliance with a eutocic vaginal delivery checklist after learning about its use in a simulation workshop attended by first-year gynaecology and obstetrics residents, concluding that the use of this type of tool serves to anticipate risk situations and reduce the number of adverse perinatal outcomes [12]. 

The aim of the Basic Principles for Clinical Gynaecological Exploration (BPCGE) Workshop is to ensure the adequate performance of a basic gynaecological examination. The key to success is not only correct technical execution but also the creation of an environment in which privacy, communication, confidentiality and respect for the patient are important [4]. These skills are reflected in the simulations to which the students are exposed in this workshop.

Medical students undergoing gynaecological examination workshops has already been assessed by other authors, such as Pugh C.M. et al. [13] from Chicago (USA) and Janjua A. et al. [4] from London (UK). Nevertheless, as far as we know, there are no references that evaluate this tool of medical training in Spain, also evaluating its impact in two consecutive academic years and following a line of semi-face-to-face work, as has been performed in the previous works of our own group [11,12]. 

For these reasons, this study was designed to evaluate whether the BPCGE, offered to medical students, improves theoretical and practical knowledge, safety, confidence, global satisfaction and the achievement of the proposed objectives in the area of gynaecological clinical examinations.

## 2. Materials and Methods

This was a pre–post-quasi-experimental study with a longitudinal follow up that started 1 week before participants attended the Basic Principles for Clinical Gynaecological Exploration (BPCGE) Workshop and up to 2 weeks afterwards.

The work was carried out in accordance with Strengthening the Reporting of Observational Studies in Epidemiology (STROBE) guidelines [14]. This workshop was offered at the Gynaecology and Obstetrics department of Hospital General Universitario Gregorio Marañón in Madrid during the 2020–2021 and 2021–2022 academic years. The study participants were 4th-year students earning a degree in Medicine and were enrolled in the Gynaecology and Obstetrics programme in one of the two academic years. The students were offered the BPCGE Workshop and voluntarily decided to attend. Participant selection was not carried out randomly because all of the intended participants were medical students.

The BPCGE Workshop is a semi-face-to-face course with an online module, which takes about 4–6 h, and a face-to-face part that lasts 2 h and in which, after an explanation of the gynaecological examination, the training is eminently practical. 

**Figure 1:**
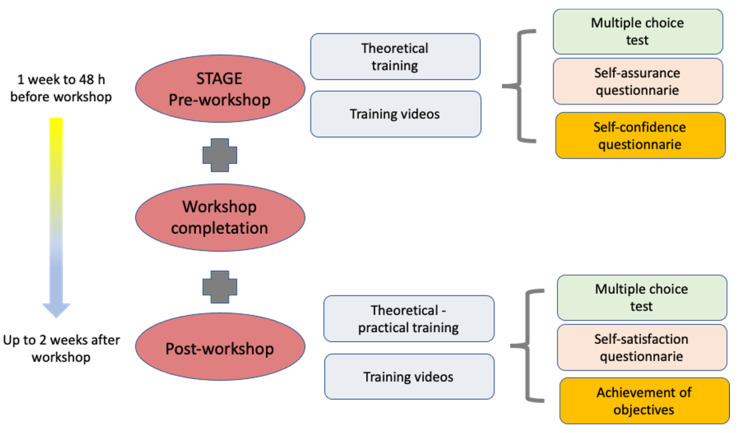
Stages and content of the Workshop on Basic Principles for Clinical Gynaecological Exploration.

The workshop was divided into three phases (Figure 1), which were adequately explained to the students. The phases began prior to the workshop (phase 1), continued during it (phase 2) and ended after (phase 3):

PHASE 1: This phase was carried out through a virtual platform (www.aleesca.es/moodle (accessed on 11 April 2023)). The students could access the “online” platform any time beginning 1 week before the face-to-face workshop up to 48 h prior to the workshop. They had access to various materials, such as explanatory documents, flyers and theoretical content (presentations, videos and images), organized with the clinical gynaecologic examination objective. The following activities were included:

A. A multiple-choice test (MCT) containing 20 questions was designed to be answered in 30 min to assess theoretical–practical knowledge pertaining to the subject. Each correct answer was worth 0.5 points (no points were subtracted for incorrect answers). The purpose of the test was to evaluate the theoretical and practical knowledge of the students on the subject prior to the workshop. All the theoretical content was specially designed by the HGUGM team of trainers with extensive teaching and clinical experience (including the most experienced obstetricians and gynaecologist in our centre) and teachers of CUM, responsible for university teaching. These professionals validated the MCTs in the previous academic courses.

B. Two self-administered questionnaires were used to assess the self-assurance and self-confidence of the students when facing a similar clinical situation. Replies were scored using a semiquantitative Likert scale [15] (Appendix A).

**Figure 2:**
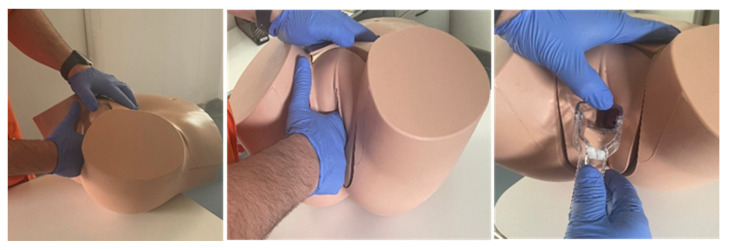
Clinical Female Pelvic Trainer Mk 3 (CFPT) mannequin, from Limbs & Things Ltd., Bristol, UK [16], an anatomical model of the female pelvis used in simulations of basic gynaecological examinations.

PHASE 2: All the students were divided into groups of 8–10 to attend the 2 h workshop in person. The workshop began with a brief presentation by residents and/or physicians of the Department of Gynaecology and Obstetrics on the subject of Basic Principles for Clinical Gynaecological Exploration. Subsequently, the presentation of said knowledge was carried out on a mannequin, simulating a probable clinical situation to demonstrate the practical skills to be developed by the students. This mannequin (Clinical Female Pelvic Trainer Mk 3 (CFPT), from Limbs & Things Ltd., Bristol, UK, [16]), shown in Figure 2, was a very precise and tactile anatomical model of the female pelvis, on which the different clinical scenarios were simulated.

To end phase 2, the group, both students and residents, met to provide feedback on the workshop. Likewise, doubts, curiosities and feelings in relation to the workshop were discussed.

PHASE 3: In the period between the end of phase 2 and up to 2 weeks after the workshops, the following were completed online:

C. An MCT similar to that in stage 1 but with questions designed to compare each student’s understanding of the topic and practical skills before and at two time points after the workshop was applied.

D. Two self-administered questionnaires designed to assess self-satisfaction and the achievement of objectives were applied only in this phase of the study (Appendix A). 

Students who did not participate in any of the workshop phases were excluded, as were repeaters in the second year because their data were included for the previous year. In addition, students who performed the assessments in less than 3 min or, conversely, in more than 30 min and those who required more than one attempt to complete them, were also excluded. To ensure that these exclusion criteria were met, the online tool recorded attendance and participation in the different phases of the workshop. In addition, this tool allowed the students to carry out each and every one of the activities in succession in an orderly manner over time. Finally, to exclude repeaters, the tool recorded the date of access to the platforms; therefore, the most recent results for the students who attended the workshop in two consecutive years were eliminated.

The variables analysed (Table 1) were student characteristics, such as gender, age, academic year in which the student was enrolled and previous passive or active experience in previous gynaecological examinations (never vs. one or more occasions). In addition, prior to the workshop and through self-administered questionnaires, the confidence (Likert scale, with values between 0 and 10 points: not at all confident (0–2), rarely confident (3–4), somewhat confident (5–6), confident (7–8) and very confident (9–10)) and self-assurance (Likert scale (0–10): poor (0–2), medium (3–4), good (5–6), very good (7–8) and excellent (9–10)) of the students were self-evaluated. The theoretical–practical knowledge of the students was analysed by performing an MCT both before and after the workshop (score of 0–10, in 0.5-point increments). Finally, after the workshop and through self-administered questionnaires, perceived satisfaction (Likert scale (0–10): totally dissatisfied (0–2), dissatisfied (3–4), neutral (5–6), satisfied (7–8) and totally satisfied (9–10), and 5 additional questions with open answers) and the achievement of the proposed objectives (Likert scale (0–10): totally not achieved (0–2), not achieved (3–4), neutral (5–6), achieved (7–8) and fully achieved (9–10), and 2 questions with open answers) were analysed.

The responses to the MCTs and questionnaires were obtained from the virtual platform and stored in several Excel sheets for an analysis. Statistical tests were performed using the software package SPSS Version 21.0 (IBM Corp., Armonk, NY, USA). Quantitative variables are expressed as the mean and standard deviation, and categorical variables are expressed as the number and percentage. During the analysis of the MCT, it was assumed that the grades obtained in both phases in the two academic years followed a normal distribution because the sample size was greater than 30 students and the mean and median were similar. For the pre–post-comparative analysis of the MCTs, Student’s *t* test was used for paired samples; for the rest of the variables, comparisons between the first and second years were carried out using Student’s *t* test for independent samples. The quantitative variables were analysed using the chi-square test. *p* < 0.05 was considered statistically significant.

## 3. Results

**Figure 3:**
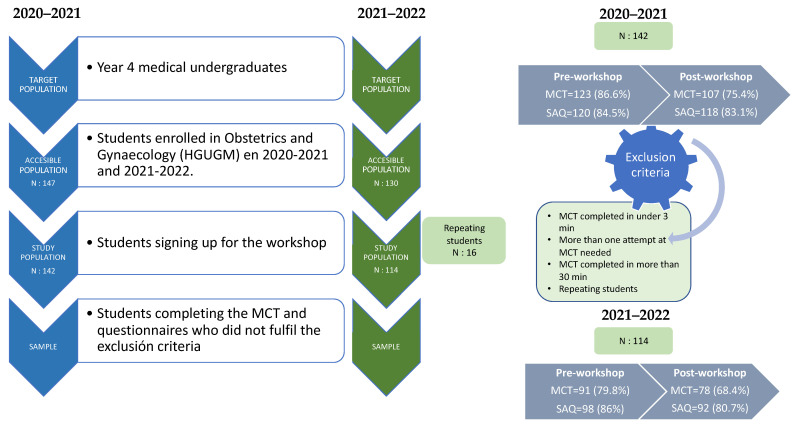
Participant recruitment.

**Figure 4:**
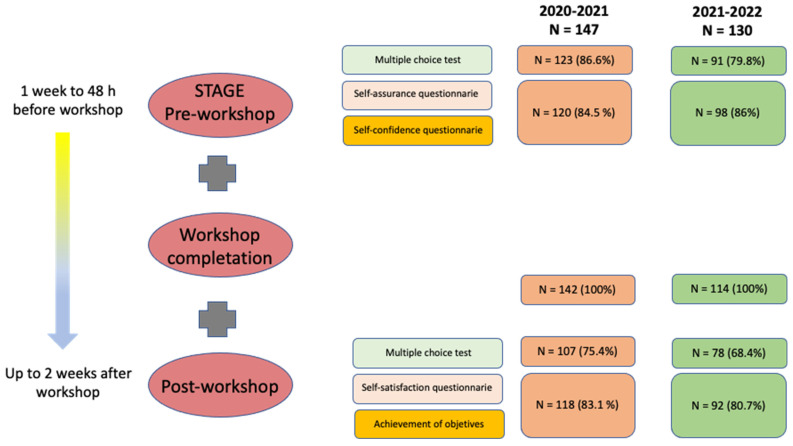
Workshop stages, tasks and participation.

During the first academic year (2020–2021), 147 students were enrolled, of whom 142 (97.2%) took part in the workshop. In the second year (2021–2022), of the 130 enrolled students, 114 (87.7%) participated in the workshop. After applying the exclusion criteria, the percentage of participation in the pre-workshop and workshop stages was high (equal to or greater than 80%), slightly decreasing for the post-workshop stage (72.8% and 68.4% for the first and second academic years, respectively, based on the completion of the second MCT) (Figure 3 and Figure 4).

The average age of the students was 21.5 ± 1.8 years for the first academic year and 21.7 ± 2.2 years for the second academic year, and the percentage of males was 34.1% and 32.7% in the first and second academic years, respectively; the differences were not statistically significant.

The clinical gynaecological examination experience of the students, both active and passive, prior to the workshop is shown in Table 2. In 2020–2021, of the 120 students who participated, only 21 (17.5%) had participated passively and 6 (5%) actively in one or more gynaecological clinical examinations. In the 2021–2022 academic year, of the 98 students who participated, 19 (19.4%) had previously participated passively and 9 (9.2%) actively in one or more gynaecological clinical examinations. There was a higher percentage of individuals with active experience in gynaecological examinations in the second year than in the first year (*p* = 0.02).

The MCT scores were high both prior to the workshop (8.19 in the first year and 7.95 in the second) and after the workshop (9.57 vs. 9.16, respectively). Between the pre-workshop and 2 weeks post-workshop stages, there was significant improvement in theoretical–practical knowledge (improvement mean = 1.38 and 1.21 in 2020–2021 and 2021–2022 academic years, respectively; *p* < 0.001) (Table 3).

The safety and confidence results for the students, measured through self-administered pre-workshop surveys, are shown in Table 4 and Table 5, respectively. The average grade for all items was less than 5 points, indicating that the students’ low security and confidence before the workshop; there were no significant differences between the two academic years.

The results for satisfaction and the achievement of objectives, measured using post-workshop self-administered surveys, are shown in Table 6 and Table 7, respectively. One hundred percent of the values are close to or exceed 8 points, indicating that overall, the students were satisfied and achieved the objectives of the workshop. Notably, the high scores in both academic years were related to the teachers of the workshop (“assesses the ease with which teachers create a climate of trust” and “assesses the ease with which teachers listen with interest to the students”), to the recommendation of other classmates to attend the course and to overall satisfaction after the completion of the workshop. However, compared with the 2020–2021 academic year, for the 2021–2022 academic year, there were statistically significant decreases in the following items of the satisfaction survey: “workshop schedule”, “duration of the workshop”, ”adaptation of the classrooms used in the workshop”, “coordination between workshop teachers”, “ease with which teachers allowed me to participate” and “ease with which teachers created a climate of trust”; there were no differences in the other items studied. However, in both academic years, students expressed their satisfaction with the BPCGE and felt as though they achieved the objectives of the workshop, confirmed through high test scores, which in some cases were outstanding.

## 4. Discussion

The results of this study indicate that with a total of 256 students and a participation rate higher than 70%, theoretical–practical knowledge significantly improved after the Workshop on Basic Principles for Clinical Gynaecological Exploration. In addition, the students expressed great satisfaction, reaching the learning objectives proposed in the workshop.

In recent years, there has been a growing number of studies that have evaluated various simulation strategies for gynaecological examinations with medical students [1,4,5,17,18]. We highlight, due to the high number of participants, a publication by Janjua A. et al. [4], with 492 final-year medical students; in comparison, our study included 256 fourth-year students earning a degree in Medicine, a difference that we believe is important because the early start of simulation strategies from the first years of training probably favours the learning of the students and their involvement in subsequent clinical rotations.

With regard to participation by gender, two out of three students were women, contrary to what was published by Pugh C.M. et al. [5], who reported that the proportion of men was higher (51.5% vs. 48.5%). However, the participation of women in health science careers is increasing. According to data published by the Spanish National Institute of Statistics, in the 2020–2021 academic year, women represented 71.8% of registered students [19] and up to 52.8% of registered doctors in Spain [20]. These results guide us to reflect on the role of traditional gender stereotypes in the choice of certain careers, such that women tend to choose those professions where care and concern for others prevail, and men maintain the choice of technical education due to better job opportunities and higher economic remuneration, recognition and social prestige [21]. 

Participation in each stage of the workshop was high (at least 70%), consistent with what was published by Janjua A. et al. [4] (83%). This finding indicates the enormous receptivity and interest shown by medical students. Our centre, Gregorio Marañón General University Hospital, receives a large number of medical students each academic year. In addition, the Obstetrics and Gynaecology department has been carrying out, for more than 5 years, a large number of clinical simulation courses, such as the Eutocic Vaginal Delivery Workshop and the Blood Loss Quantification in Obstetrics Workshop. All workshops have been regarded as positive and useful tools by the students, a fact that has probably had an impact on the high number of participants who voluntarily signed up for the BPCGE.

Regarding the clinical experience of the students, there were significant differences between the two academic years, with active participation in a gynaecological examination being greater in the second year than in the first. This may be because during the first year of the study, there were greater restrictions in clinical practice due to the COVID-19 pandemic [22], which led students to have fewer opportunities to obtain practical experience. We argue that taking the BPCGE workshop during higher-level years and, more specifically, while studying gynaecology and obstetrics, as is the case for 4th-year medical students, could be favourable because these students have a greater possibility of putting into practice the skills and knowledge obtained during the workshop in a real situation, as they will soon enter clinical practice.

The MCT results regarding the acquisition of theoretical–practical knowledge were high, both prior to the workshop and after its completion, increasing significantly by 1.4 and 1.2 points for the first and second academic years, respectively. These findings are consistent with results reported in the literature, as any educational intervention should result in an increase in students’ knowledge base [23]. This increase may seem unsurprising. Although this small difference may not be important, it must be considered that the students had high scores during the different stages of the workshop (8–9 out of 10). We must assess, in future editions of the BPCGE workshop, whether, by changing the methodology, type or difficulty of the questions asked before the workshop, the impact of training in this field can be better assessed.

The results obtained from the self-administered surveys showed the low level of confidence and security of the students before the workshop (the average score for all items was less than 5 points). Although the analysis of the impact of simulation training on participant confidence has shown mixed results [24,25], there is still a need to carry out these types of training workshops. With simulation, it is possible to improve the knowledge and skills of students and to promote interprofessional communication and communication with patients, thus increasing the security, confidence and self-sufficiency of students when interacting with and treating patients [26,27]. 

The post-workshop satisfaction and goal achievement scores were significantly higher than the pre-workshop satisfaction and goal achievement scores, as all scores approached or exceeded 8 points. Other studies have analysed student satisfaction, determining that simulation-based learning methods favour student knowledge and satisfaction [7,11,28,29]. Although satisfaction is not a guarantee of learning, the positive reaction of students, measured with satisfaction surveys, is the first criterion for evaluating education and training programmes [10]. The next level of assessment, that of learning and the knowledge acquired by students, was also conducted, both objectively, with improvements in the post-workshop MCT scores, and subjectively, with the high scores obtained for the achievement of objectives survey. Therefore, the increase in knowledge, improvements in the practical skills and motivation of the students will probably result in greater training and safety when caring for real patients [30].

The teachers of the workshop received the highest scores possible from the students in the satisfaction surveys in both academic years. Teachers in simulation play a significant part in the successful implementation of simulation programmes because they are valuable gatekeepers of evidence-based knowledge and partners in leadership for educational issues [27,31]. Therefore, we believe that the teachers of the workshop have more than achieved the proposed objectives because they created a climate of trust during the workshop and facilitated open communication, thus improving the effectiveness of teamwork.

Among the strengths of this study are the large number of students in all phases of the workshop and the excellent response shown by the students during the study period. Likewise, the results achieved were homogeneous during the two consecutive academic years.

Regarding the limitations, analyses of the confidence and security of the students were only performed before the workshop and not after it, as has been suggested in the work published by Ruiz-Labarta J. et al. [11]. Therefore, it is not possible to assess the evolution of these items after the course. In addition, the long-term changes in the behaviour of the students were not analysed, as follow-up surveys were carried out only 2 weeks after attending the workshop.

## 5. Conclusions

Our students, after outstanding participation, evaluated the BPCGE, and improved their theoretical and practical knowledge, as well as their skills in a gynaecological clinical examination. Moreover, in their view, after the workshop, they felt very satisfied, far outreaching the proposed aims. This study emphasizes the potential of virtual clinical simulations, in which students can hone their abilities in a secure setting without running the risk of endangering actual patients. They can repeat steps until they master them by making mistakes, learning from them and doing so again.

Therefore, these simulation workshops should be included in practical training curriculum in the future. In this way, students will acquire clinical and doctor-patient relationship skills in the year prior to clinical rotations. This training is more relevant in specialties as sensitive as gynaecology and obstetrics.

## Figures and Tables

**Table 1:** List of study variables and assessment methods and measures.

Variable	Assessment Method	Qualifier
Sex	Student characteristic	Qualitative nominal
Age	Student characteristic	Quantitative discrete
Academic course	Student characteristic	Qualitative ordinal
Previous passive or active experience in gynecological examination	Student characteristic	Quantitative discrete 0 occasions; ≥1 occasion
Self-confidence	Self-administered questionnaire	Quantitative discrete Likert scale (0–10) Not at all confident (0–2), Rarely confident (3–4), Somewhat confident (5–6), Confident (7–8) and Very confident (9–10)
Self-assurance	Self-administered questionnaire	Quantitative discrete Likert scale (0–10) Poor (0–2), Medium (3–4), Good (5–6), Very good (7–8) and Excellent (9–10)
Theoretical-practical knowledge	Multiple choice test	Quantitative discrete Score of 0–10 in 0.5-point steps
Satisfaction	Self-administered questionnaire	Quantitative discrete Likert scale (0–0). Totally dissatisfied (0–2), Dissatisfied (3–4), Neutral (5–6), Satisfied (7–8) and Totally satisfied (9–10)
Achievement of objetives	Self-administered questionnaire	Quantitative discrete Likert scale (0–10) Totally not achieved (0–2), Not achieved (3–4), Neutral (5–6), Achieved (7–8) and Fully achieved (9–10)

**Table 2:** Clinical experience of the students at the beginning of the study.

	2020–2021 Pre-Workshop N = 120		2021–2022 Pre-Workshop N = 98	
	0 Chances	≥1 Chances	0 Chances	≥1 Chances
Passively assisted gynecological examinations	99 (82.5%)	21 (17.5%)	79 (80.6%)	19 (19.4%)
Actively assisted gynecological examinations	114 (95%)	6 (5%)	89 (90.8%)	9 (9.2%)

**Table 3:** MCT results and improvements for the two academic years.

Results of Multiple Choice Test				
	**Pre-Workshop** **N = 97**	**Post-Workshop** **N = 97**	**Improvement (μ Post—μ pre)** **N = 107**	***p***
**2020–2021 Score (/10)**	8.19 ± 1.13	9.57 ± 0.68	1.38 ± 1.07	<0.001
	**Pre-Workshop** **N = 67**	**Post-Workshop** **N = 67**	**Improvement (μ Post—μ pre)** **N = 78**	
**2021–2022 Score (/10)**	7.95 ± 0.89	9.16 ± 0.09	1.21 ± 1.08	<0.001

**Table 4:** Results of the self-assurance questionnaire for the two academic years.

Self-Assurance Questionnaire			
	2020–2021 Pre-Workshop N = 120	2021–2022 Pre-Workshop N = 98	*p*
Before a gynecological examination	3.78 ± 2.27	3.41 ± 1.94	0.199
Control and insert the speculum	3.04 ± 2.31	3 ± 2.25	0.893
Assessment of the cervix	3.07 ± 2.21	3.14 ± 1.98	0.789
Performing an examination with the supervision of a consultant	4.78 ± 2.26	4.67 ± 2.40	0.75
Performing an examination without the supervision of a consultant	2.44 ± 2.05	2.63 ± 1.98	0.486

**Table 5:** Results of the self-confidence questionnaire for the two academic years.

Self-Confidence Questionnaire			
	2020–2021 Pre-Workshop N = 120	2021–2022 Pre-Workshop N = 98	*p*
Theoretical basic gynecological examination	4.17 ± 2.29	3.94 ± 2.09	0.445
Knowledge about the basic concepts	4.23 ± 2.32	4.15 ± 2.25	0.796
Practical basic gynecological examination	3.28 ± 2.21	3.38 ± 2.00	0.706

**Table 6:** Results of the self-satisfaction questionnaire for the two academic years.

Self-Satisfaction Questionnaire			
	2020–2021 Post-Workshop N = 116	2021–2022 Post-Workshop N = 92	*p*
Previous information of the course (in reference to the publicity of the same)	7.97 ± 1.80	8.27 ± 1.48	0.192
Evaluate the documentation provide during the course	8.30 ± 1.43	8.45 ± 1.40	0.469
Evaluate the organization of the course	8.99 ± 1.11	8.88 ± 0.98	0.447
Evaluate the timetable of the course	8.79 ± 1.20	8.03 ± 1.69	<0.001
Evaluate the duration of the course	8.97 ± 1.16	8.59 ± 1.26	0.024
Evaluate the adequacy of the course classrooms	9.21 ± 0.93	8.92 ± 1.04	0.044
Evaluate the adequacy and quality of the practical or didactic material of the course	8.97 ± 1.13	8.88 ± 1.33	0.589
Evaluate the teaching capacity of the course teachers	9.48 ± 0.79	9.30 ± 0.85	0.131
Evaluate the coordination between the course teachers	9.42 ± 0.79	9.13 ± 0.95	0.02
Evaluate the coordination between theoretical and practical contents of the course	8.69 ± 1.20	8.76 ± 1.03	0.658
Evaluate the ease with which teachers allow participation	9.55 ± 0.71	9.33 ± 0.81	0.045
Evaluate the ease with which teachers listen with interest to students	9.56 ± 0.69	9.38 ± 0.82	0.08
Evaluate the ease with which teachers create a climate of trust	9.60 ± 0.71	9.30 ± 0.90	0.007
I have received information about the general aims of the course	9.07 ± 0.97	8.99 ± 1.14	0.596
The course has achieved the proposed aims	9.08 ± 0.94	9.02 ± 0.85	0.657
The contents of the course have corresponded to what was expected when you enrolled in the course	8.97 ± 1.07	9.00 ± 0.95	0.855
The level of knowledge with which the topics have been dealt with has been adequate	9.16 ± 0.87	9.15 ± 0.85	0.98
Do you consider that the course is of interest for your professional activity?	9.30 ± 0.90	9.37 ± 0.91	0.586
Would recommend attending this course to your colegues?	9.42 ± 0.84	9.40 ± 0.77	0.882
Are you satisfied that you have taken this course?	9.42 ± 0.85	9.40 ± 0.74	0.881

**Table 7:** Results of the achievement of objectives for the two academic years.

Achievement of Objetives Questionnaire			
	2020–2021 Post-Workshop N = 116	2021–2022 Post-Workshop N = 92	*p*
I have received information about the general aims of the course	9.18 ± 0.90	8.96 ± 1.30	0.16
After the workshop I have reached the aim “Know and identify the key points in the assessment of the clinical history in gynecology”“	8.82 ± 1.04	8.87 ± 1.03	0.733
After the workshop I have reached the aim “Know and perform a basic gynecological examination (visualization and vaginal touch)”	9.04 ± 0.95	9.00 ± 0.98	0.752
After the workshop I have reached the aim “Know and perform a basic gynecological examination (bimanual touch)”	8.81 ± 1.18	8.92 ± 1.20	0.501
After the workshop I have reached the aim “Know and understand the key points of basic gynecological examination (speculoscopy and cytology)”	9.02 ± 0.87	8.88 ± 1.07	0.309
After the workshop I have reached the aim “Know and perform a speculoscopy and the key points to perform a vaginal cytology”	8.88 ± 0.89	8.87 ± 1.02	0.936

## Data Availability

The data used to support the findings of the present study are available from the corresponding author upon request.

## Appendix A. Self-Confidence, Self-Assurance, Self-Satisfaction and Achievement of Objectives Questionnaires

**Self-Confidence Questionnaire**					
**Question**	**Reply**				
Theoretical basic gynecological examination	Not at all confident (0–2)	Rarely confident (3–4)	Somewhat Confidence (5–6)	Confident (7–8)	Very confident (9–10)
Knowledge about the basic concepts	Not at all confident (0–2)	Rarely confident (3–4)	Somewhat Confidence (5–6)	Confident (7–8)	Very confident (9–10)
Practical basic gynecological examination	Not at all confident (0–2)	Rarely confident (3–4)	Somewhat Confidence (5–6)	Confident (7–8)	Very confident (9–10)

**Self-Assurance Questionnaire**					
**Questio** **n**	**Reply**				
Before a gynecological examination	Poor (0–2)	Medium (3–4)	Good (5–6)	Very good (7–8)	Excellent (9–10)
Control and insert the speculum	Poor (0–2)	Medium (3–4)	Good (5–6)	Very good (7–8)	Excellent (9–10)
Assessment of the cervix	Poor (0–2)	Medium (3–4)	Good (5–6)	Very good (7–8)	Excellent (9–10)
Performing an examination with the supervision of a consultant	Poor (0–2)	Medium (3–4)	Good (5–6)	Very good (7–8)	Excellent (9–10)
Performing an examination without the supervision of a consultant	Poor (0–2)	Medium (3–4)	Good (5–6)	Very good (7–8)	Excellent (9–10)

**Self-Satisfaction Questionnaire**					
**Question**	**Reply**				
Previous information of the course (in reference to the publicity of the same)	Totally dissatisfied (0–2)	Dissatisfied (3–4)	Neutral (5–6)	Satisfied (7–8)	Totally satisfied (9–10)
Evaluate the documentation provide during the course	Totally dissatisfied (0–2)	Dissatisfied (3–4)	Neutral (5–6)	Satisfied (7–8)	Totally satisfied (9–10)
Evaluate the organization of the course	Totally dissatisfied (0–2)	Dissatisfied (3–4)	Neutral (5–6)	Satisfied (7–8)	Totally satisfied (9–10)
Evaluate the timetable of the course	Totally dissatisfied (0–2)	Dissatisfied (3–4)	Neutral (5–6)	Satisfied (7–8)	Totally satisfied (9–10)
Evaluate the duration of the course	Totally dissatisfied (0–2)	Dissatisfied (3–4)	Neutral (5–6)	Satisfied (7–8)	Totally satisfied (9–10)
Evaluate the adequacy of the course classrooms	Totally dissatisfied (0–2)	Dissatisfied (3–4)	Neutral (5–6)	Satisfied (7–8)	Totally satisfied (9–10)
Evaluate the adequacy and quality of the practical or didactic material of the course	Totally dissatisfied (0–2)	Dissatisfied (3–4)	Neutral (5–6)	Satisfied (7–8)	Totally satisfied (9–10)
Evaluate the teaching capacity of the course teachers	Totally dissatisfied (0–2)	Dissatisfied (3–4)	Neutral (5–6)	Satisfied (7–8)	Totally satisfied (9–10)
Evaluate the coordination between the course teachers	Totally dissatisfied (0–2)	Dissatisfied (3–4)	Neutral (5–6)	Satisfied (7–8)	Totally satisfied (9–10)
Evaluate the coordination between theoretical and practical contents of the course	Totally dissatisfied (0–2)	Dissatisfied (3–4)	Neutral (5–6)	Satisfied (7–8)	Totally satisfied (9–10)
Evaluate the ease with which teachers allow participation	Totally dissatisfied (0–2)	Dissatisfied (3–4)	Neutral (5–6)	Satisfied (7–8)	Totally satisfied (9–10)
Evaluate the ease with which teachers listen with interest to students	Totally dissatisfied (0–2)	Dissatisfied (3–4)	Neutral (5–6)	Satisfied (7–8)	Totally satisfied (9–10)
Evaluate the ease with which teachers create a climate of trust	Totally dissatisfied (0–2)	Dissatisfied (3–4)	Neutral (5–6)	Satisfied (7–8)	Totally satisfied (9–10)
I have received information about the general aims of the course	Totally dissatisfied (0–2)	Dissatisfied (3–4)	Neutral (5–6)	Satisfied (7–8)	Totally satisfied (9–10)
The course has achieved the proposed aims	Totally dissatisfied (0–2)	Dissatisfied (3–4)	Neutral (5–6)	Satisfied (7–8)	Totally satisfied (9–10)
The contents of the course have corresponded to what was expected when you enrolled in the course	Totally dissatisfied (0–2)	Dissatisfied (3–4)	Neutral (5–6)	Satisfied (7–8)	Totally satisfied (9–10)
The level of knowledge with which the topics have been dealt with has been adequate	Totally dissatisfied (0–2)	Dissatisfied (3–4)	Neutral (5–6)	Satisfied (7–8)	Totally satisfied (9–10)
Do you consider that the course is of interest for your professional activity?	Totally dissatisfied (0–2)	Dissatisfied (3–4)	Neutral (5–6)	Satisfied (7–8)	Totally satisfied (9–10)
Would recommend attending this course to your colegues?	Totally dissatisfied (0–2)	Dissatisfied (3–4)	Neutral (5–6)	Satisfied (7–8)	Totally satisfied (9–10)
Are you satisfied that you have taken this course?	Totally dissatisfied (0–2)	Dissatisfied (3–4)	Neutral (5–6)	Satisfied (7–8)	Totally satisfied (9–10)

**Achievement of Objetives Questionnaire**					
**Question**	**Reply**				
I have received information about the general aims of the course	Totally not achieved (0–2)	Not achieved (3–4)	Neutral (5–6)	Achieved (7–8)	Fully achieved (9–10)
After the workshop I have reached the aim “Know and identify the key points in the assessment of the clinical history in gynecology”	Totally not achieved (0–2)	Not achieved (3–4)	Neutral (5–6)	Achieved (7–8)	Fully achieved (9–10)
After the workshop I have reached the aim “Know and perform a basic gynecological examination (visualization and vaginal touch)”	Totally not achieved (0–2)	Not achieved (3–4)	Neutral (5–6)	Achieved (7–8)	Fully achieved (9–10)
After the workshop I have reached the aim “Know and perform a basic gynecological examination (bimanual touch)”	Totally not achieved (0–2)	Not achieved (3–4)	Neutral (5–6)	Achieved (7–8)	Fully achieved (9–10)
After the workshop I have reached the aim “Know and understand the key points of basic gynecological examination (speculoscopy and cytology)”	Totally not achieved (0–2)	Not achieved (3–4)	Neutral (5–6)	Achieved (7–8)	Fully achieved (9–10)
After the workshop I have reached the aim “Know and perform a speculoscopy and the key points to perform a vaginal cytology”	Totally not achieved (0–2)	Not achieved (3–4)	Neutral (5–6)	Achieved (7–8)	Fully achieved (9–10)
